# The Foster-Brothers

**Published:** 1881-02

**Authors:** 


### THE FOSTER-BROTHERS.

IT WAS on an April morning that Mr. Cade Marshall, having nothing else to do for the moment, stood on his door-step and looked at the Mississippi River.

It was not many years ago—yet since then a thousand glories and shames have dazzled and affronted the world ; myriads of bright things have been darkened, and dark things brought to light ; a continent has been dipped in blood, and has arisen from the red baptism cleansed of its deadliest sin. There is not a man now living who has precisely the same political ideas which he had on that April morning when Mr. Cade Marshall, idly enjoying the spring sunshine, looked from the door-step of his house on an Illinois bluff at the great river, and over it to the Missouri shore.

Mr. Marshall had been a Bachelor of Arts half a year. He had spent this time at home in the city of Moscow, Illinois, an ambitious town that had stretched itself so far over the hills and hollows skirting the river that it seemed doubtful whether it would ever knit its overgrown members firmly together. It had cut one hill in two, in the hope of a bridge which never was built. It had filled up one ravine, in preparation for a railroad which never arrived. It had a special charter from the Legislature, in case it should ever be big enough to need a city government ; and it embraced several miles of the adjoining country within its corporate limits, to get taxes enough to keep up the expense of fire and lights for its Common Council. With its four or five thousand inhabitants it occupied about as much room as Paris, and sprawled over its half-dozen hills—as the elder Marshall once observed—"like a small but conceited hen trying to hatch a square yard of eggs."

A Norse poet mentions, as among the prerogatives of the gods, that they always look down. So the city of Moscow, sprinkled over its ragged bluffs, enjoyed much substantial comfort in looking across the river to where the city of Thebes clung with a precarious foothold to the Missouri mud—only existing by sufferance of the great river.

It was with a certain comfortable sense of superiority to fluvial accidents that Cade Marshall walked to his gate and glanced down the steep hill-path, two hundred feet fall to the water-side.

"The river is certainly rising," he thought, "and yet the *Lucy*

*Bertram* seems to be stuck to the landing." But the steamer, which had been blowing and whistling, and ringing bells, and stirring up the yellow sand with her revolving paddles, now swung loose and headed for the Illinois shore, dancing coquettishly sideways over the water, keeping her head up stream.

Two or three men with revolvers in their hands went shuffling by the gate.

"Thar she blows. Hurry up! Hi! or you won't fetch her."

"You bet I fetch her," answered a tall man with blue goggles. "Mornin,' Mr. Marshall! Come along and help tie up the *Lucy*."

Mr. Marshall, much amused at being thus suddenly enrolled in the constable's *posse*, followed that functionary and said, "What has she been guilty of, Captain Ketchum?"

"Why, Jim Whaler missed his carpet-bag as he was a-comin' up from St. Louis, and he swears he believes the cap'n stoled it. I reckon he never done it; but that ain't my business. The cap'n offered to compermise by payin' for what was into it. So Jim he drawed out a list: one bowie-knife, one plug o' terbacker, one deck o' keerds, and two shirts. When the cap'n seed that he jest sung out, 'Oh, gas! Two shirts! when did you ever git two shirts?' So Jim he's got his back up, and he's took out a 'tachment, and we're goin' down to tie up the *Lucy* 'till they pay."

"Did you have the shirts, Jim?" said Marshall to the injured Whaler.

"I will, 'fore I'm done with 'em."

They reached the landing just as a deck hand came ashore to cast off the cable.

"Hold on there, my African brother," shouted Ketchum. The sulky Whaler stood by the rope, while the constable went on board and served process. Marshall went with him. As they reached the bar-room a youthful figure started up from near the stove, and a clear, hearty voice shouted, "Bless your dissolute heart, Occidental! how are you?"

Marshall started at the familiar college nick-name, and turning, saw his friend and class mate, Clarence Brydges.

"*A la bonne heure!* Where is your luggage? Why didn't you tell me you were coming?"

"I am not coming. I am going to St. Paul."

"St. Paul can wait. Your boat is tied up. The captain and our constable will quarrel all day. You must go home with me.

Give me your checks. 'False, fleeting, perjured Clarence,' to slip by without ungirding yourself beneath my roof-tree."

Brydges was soon convinced by the highly-seasoned discourse of the captain that his friend had spoken the truth. They went on shore, and Marshall called a broad-shouldered, dwarfish German boy.

"Chris, take this trunk to my father's. Where is your cart?"

"I don't got none a'ready. Hans Doppelfritz his stief-father gone dead directly, und mine gart is a funeral. I pack him selbst."

He seized the heavy trunk and trotted up the steep hill-side like a mountain-goat.

Marshall and Brydges were preparing to follow, when Whaler rushed up, all the sulkiness gone from his hang-dog face.

"The cap'n's compermised. He agrees to pay for one shirt and treat the crowd. Come, take a drink, gentle*men*."

At that moment a negro came running off the boat with a shabby carpet-bag in his hand. Whaler saw him, and grew sulkier than ever. He took the thin, leprous-looking bag of black oil-cloth from the porter, who bowed and grinned, vainly expectant of backshish, and slunk away muttering unorthodox expressions in regard to his "misfortnit luck." He disappeared up the sunken road to the town, followed by Ketchum and his friends, who made frequent and jeering reference to "them shirts."

The steamer, after expressing by emphatic growling and puffing her indignation at the "law's delay," went on her way up the river. The friends slowly ascended the hill to Marshall's house. This was a large, rambling structure, originally built for the blockhouse of Fort Johnstone in the early Indian wars, with numerous additions and changes that had completely transformed it into a comfortable modern residence—as comfort is understood in the West—something very different from the Sybarite luxury of Fifth Avenue or the Back Bay.

"I am not sorry this happened," said Brydges. "There is no real occasion for me to hurry to St. Paul. I am making a rapid tour at the request of my father through the North. I have been reading some law in Mobile this winter, and the governor wants me, before beginning to practice, to see something of your country. You know he is a little *tête-montée* on this secession question. He thinks you will be a foreign nation in a few years, and he is anxious that I should see something of the present *régime*."

"Very well. Stay here and see it."

"But he insists on my passing all my time at representative places. St. Paul, as a northwest bastion of your power ; Chicago, the home of the gnomes—the supernatural workers ; Boston, your lighthouse ; and New York, your 'ventral ganglion.' I have his positive commands against stopping for a day any where else."

"Except in case of accidents. We will prepare a new one every day until we fill a chapter, which we will send to your respected ancestor with our dutiful regards."

Coming to the house they found Mr. Marshall the elder sitting in his easy-chair on the long veranda. A fresh, rosy, black-haired old gentleman, who could even yet break a colt, or crack walnuts with his fingers.

"You are heartily welcome, Mr. Brydges. Don't stare ; there is no second-sight in my knowing your name. That little Kobold Chris has come with your trunk, and I have sent it to your room. Will you go in, or stay here ? When you are my age you will seize every moment of such lovely weather, and keep where there is most of it."

The young men brought chairs and sat in the soft spring sunshine. The impulse of awakening life was faintly visible on the bluffs, where the dry grass began to show an under-tinge of green. The warm light lay richly on the broad river and the brown leafless-wooded islands, and touched softly in the blue distance the high hills beyond the Missouri flats.

Brydges, who was looking at the town of Thebes, which lies in the delta of the Des Moines and Mississippi rivers, said, suddenly,

"What a quantity of ponds there are in that town !"

"Ponds that have come there since morning," answered Colonel Marshall quietly. "If the river keeps its present mood it wil sponge that town away in a few days."

"If you would like to see the village before the catastrophe we will go over after dinner," said Cade, laughing. "My father has so often prophesied the damp bad end of Thebes that we have come to regard him as a Muscovite Cassandra."

The Marshalls dined at the orthodox Illinois hour of one. Mrs.

Marshall received her guest with the simplest courtesy, and made him feel instantly at home.

"I am never quite happy," she said, "when my table is three-sided. So you must keep that place, Mr. Brydges, until you are relieved."

The young men went down to the ferry in the afternoon and crossed over to Thebes. The river was tawny with mud and filled with the varied drift of the northern forests.

"The river is still on the rise, Captain?" said Marshall to the skipper of the *Osprey*, a long, silent, ruminant man.

"She's jest a-boomin," said Captain Apple, increasing the volume of the stream by about a gill of nicotized saliva. "Five inches yisterday, and the big end o' that sence mornin.' The Dessmine is worse yet. Ef I was a rat in a cellar, I'd move up garret about these here times."

The *Osprey* came to the wharf, which had almost disappeared beneath the encroaching river. With that obstinate unbelief of the disagreeable that has been given us doubtless to prevent our suffering misfortunes in anticipation, the dealers in cord-wood were busy in removing large quantities of it from the water-line, and piling it a few feet further from the shore—to be moved again next day.

Marshall and Brydges walked through the town. It had been built before the levee, and so was on an average several feet lower. The side-streets and back-yards were therefore already invaded by the waters. A great quantity had come in during the night, creeping over the low banks of the Des Moines, and attacking the town in its unleveed and therefore defenseless rear. Many flat gardens and hollow commons were suddenly filled up with the muddy flood, as if it had soaked through the thin soil from below. A good many houses were built, with a sort of make-shift fore-sight, on detached piles. These stood clear for the present from the wet, looking like slatternly women holding up their draggled skirts. One dreary frame-house they saw where the piles had given way at one end, and the house stood helplessly with one corner in the air and one in the slough. They had the indiscretion to look in at the window nearest the road, and saw a sallow woman frying bacon at a stove lashed to the wall, and some ragged urchins in high glee climbing the sloping floor like flies, and sliding down again like muskrats.

Every where a dismal air of make-shift. All the gates were tied up with ropes—the latches all gone. At the frontdoors of several rather ambitious-looking houses a small ladder supplied the place of a porch. Many of the houses were unpainted, and looked prematurely old and shabby. Every thing seemed to say, "What is the use?" Dirty clay-colored curs lounged on the muddy door-steps with a dispirited and dejected air. The very streets, that started with fair prospects, seemed to grow discouraged and to flatten supinely out into bottomless black mud. The cats found it difficult to make their visits with any regard to neat feet. Long, gaunt, red-haired hogs grunted unsociably in the dry spots that were yet left them, too listless to be hungry.

In the best quarter of the town the two friends came to a large barn-like church with an unfinished steeple, around which the scaffolding was falling to pieces. Here the sidewalk was elevated upon poles to the level of the fence-posts. This had been done some years before in the stress of former floods, and no one had as yet had energy enough to take it down. Turning its corner they found themselves before a larger and better house than any they had before seen. The garden before the door was completely submerged. A young girl standing upright in a light skiff sculled it dexterously about the garden with a long oar. It was a very pretty picture—the exquisite form swaying to every movement of the frail boat, the warm sunshine touching with gold lights the dark brown hair.

"Who has not heard of a jolly young waterman?"

sang Marshall.

She turned, and with one stroke of the oar brought her skiff to the gate; she gave her hand to Marshall with a gay "Good morning."

"Miss Des Ponts, let me present my friend, Mr. Brydges."

"Will you tempt the dangers of the deep and come in?" she said.

Marshall looked at Brydges, who eagerly nodded assent.

"You will come in first, Mr. Brydges," said the fair mariner. "Mr. Marshall is *chez lui* in my boat."

She gave Brydges her hand to assist him into the boat. It was a soft white hand—"the hand of a marquise," Balzac would have said—with a firm, vigorous grasp. Arriving at the door-step, she stepped lightly out of the skiff, and led her visitors into a cheerful-looking drawing-room carpeted in warm, bright colors, richly furnished and curtained, where a brisk fire of hickory logs cracked and sparkled in the wide chimney.

"A fancy of papa's," said Miss Des Ponts. "He insists upon this open fire until the first of May, even if we have a torrid April like this. Cade, open the windows."

Mr. Marshall obeyed the peremptory order; then, in the same familiar tone, said, "Mimi, when are you coming over to spend the forty diluvial days and nights?"

"Silence, rash Muscovite! The river is merely performing its fertilizing office for the city of its love. It will be in its bed to-morrow."

"And to-morrow, and to-morrow," added Marshall, tragically.

"I am ashamed to own," said Miss Des Ponts, "that papa has been carried away by the prevailing stampede. He wanted the furniture moved upstairs yesterday, but I fought hard and got a reprieve till to-day. I thought it would be a sort of treason to the river to distrust its honorable intentions."

"Pray, let us hear your piano once more before it is banished to the attic."

She went to the instrument, and her fingers strayed for a moment over the keys, "building a bridge from dream-land." She then played with singular feeling and expression a low, solemn, dirge-like movement, which neither of the gentlemen recognized, but which was intensely thrilling and saddening. It closed with a sudden and startling discord, and she instantly broke into one of the younger Strauss' most Champagny mazurkas, which she gave with such grace and spirit that Marshall vowed he could see the flash of white satin boots, and catch the distant popping of corks in the supper-room.

Brydges, who had been somehow vaguely annoyed at the easy familiarity existing between Marshall and Miss Des Ponts, had taken no part in the conversation. While she played he devoured her with his eyes. If she seemed lovely in the broad light outside, she was vastly more so now; her brown eyes softened by feeling, her exquisite lips slightly parted, a delicate tinge hovering like the first flush of dawn on the perfect pale cheek.

(TO BE CONTINUED.)

---

ELDERLY gentleman to a freshman on the train: "You don't have any ticket?" "No, I travel on my good looks." "Then,' after looking him over, "probably you ain't going very far."

